# Correction: Yang et al. The Expression Pattern of tRNA-Derived Small RNAs in Adult *Drosophila* and the Function of *tRF-Trp-CCA-014-H3C4* Network Analysis. *Int. J. Mol. Sci.* 2023, *24*, 6169

**DOI:** 10.3390/ijms262110544

**Published:** 2025-10-30

**Authors:** Deying Yang, Feng Xiao, Ya Yuan, Jiamei Li, Siqi Wang, Xiaolan Fan, Qingyong Ni, Yan Li, Mingwang Zhang, Xiaobin Gu, Taiming Yan, Mingyao Yang, Zhi He

**Affiliations:** 1 College of Animal Science and Technology, Sichuan Agricultural University, Chengdu 611130, China; deyingyang@sicau.edu.cn (D.Y.); xiaofengsicau@163.com (F.X.); yuanya145@163.com (Y.Y.); ljm13419358056@163.com (J.L.); wangwangsiqi@126.com (S.W.); xiaolanfan@sicau.edu.cn (X.F.); niqingyong@hotmail.com (Q.N.); liyan@sicau.edu.cn (Y.L.); mwzhangkiz@hotmail.com (M.Z.); yantaiming@sina.com (T.Y.); 2 Farm Animal Genetic Resources Exploration and Innovation Key Laboratory of Sichuan Province, Sichuan Agricultural University, Chengdu 611130, China; 3 College of Veterinary Medicine, Sichuan Agricultural University, Chengdu 611130, China; guxiaobin198225@126.com

In the original publication [1], there was a mistake in Figure 3. Upon carefully examining our original experimental results, in Figure 3F (the expression level of *tRF-Trp-CCA-014* in different tissues of mice), the marks on the two time points were reversed. The 19-month-old mice should be reversed with 5-month-old young mice in Figure 3F. The corrected Figure 3 appears below. We confirm that the scientific conclusions are unaffected. This correction was approved by the Academic Editor. The original publication has also been updated.

## Figures and Tables

**Figure 3:**
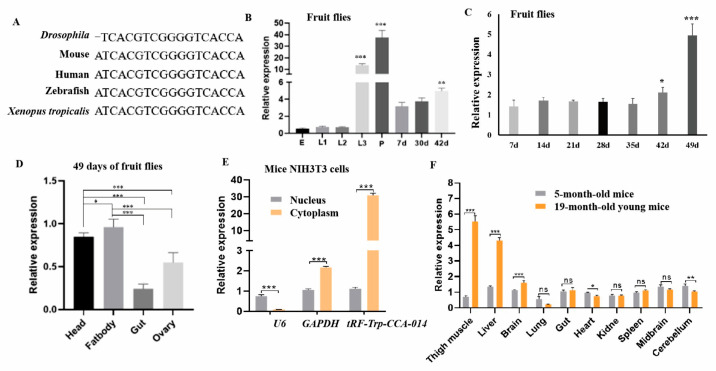
Expression features of *tRF-Trp-CCA-014* in *Drosophila* and mice. (**A**) The sequence alignment of *tRF-Trp-CCA-014* in *Drosophila*, mouse (a relational database of transfer RNA related fragments 3002a, tRFdb 3002a), human (from MINTbase v2.0), zebrafish (tRFdb 3038), and *Xenopus tropicalis* (tRFdb 3015); (**B**) the expression level of *tRF-Trp-CCA-014* in larvae and adults; (**C**) the expression level of *tRF-Trp-CCA-014* in more adult ages; (**D**) the expression level of *tRF-Trp-CCA-014* in 49-day-old fruit flies; (**E**) the expression level of *tRF-Trp-CCA-014* in the nucleus and cytoplasm of mouse NIH3T3 cells; (**F**) the expression level of *tRF-Trp-CCA-014* in different tissues of mice. E—egg; L1—the first instar larva; L2—the second instar larva; L3—the third instar larva; P—pupa; d—days. *, *p* < 0.05; **, *p* < 0.01; ***, *p* < 0.001; ns—no significance.
