# Supplementary figures and images for: The Expression Pattern of tRNA-Derived Small RNAs in Adult Drosophila and the Function of tRF-Trp-CCA-014-H3C4 Network Analysis
Source: Int J Mol Sci. 2023 Mar 24;24(7):6169. doi: 10.3390/ijms24076169 (PMC10094720; doi:10.3390/ijms24076169)

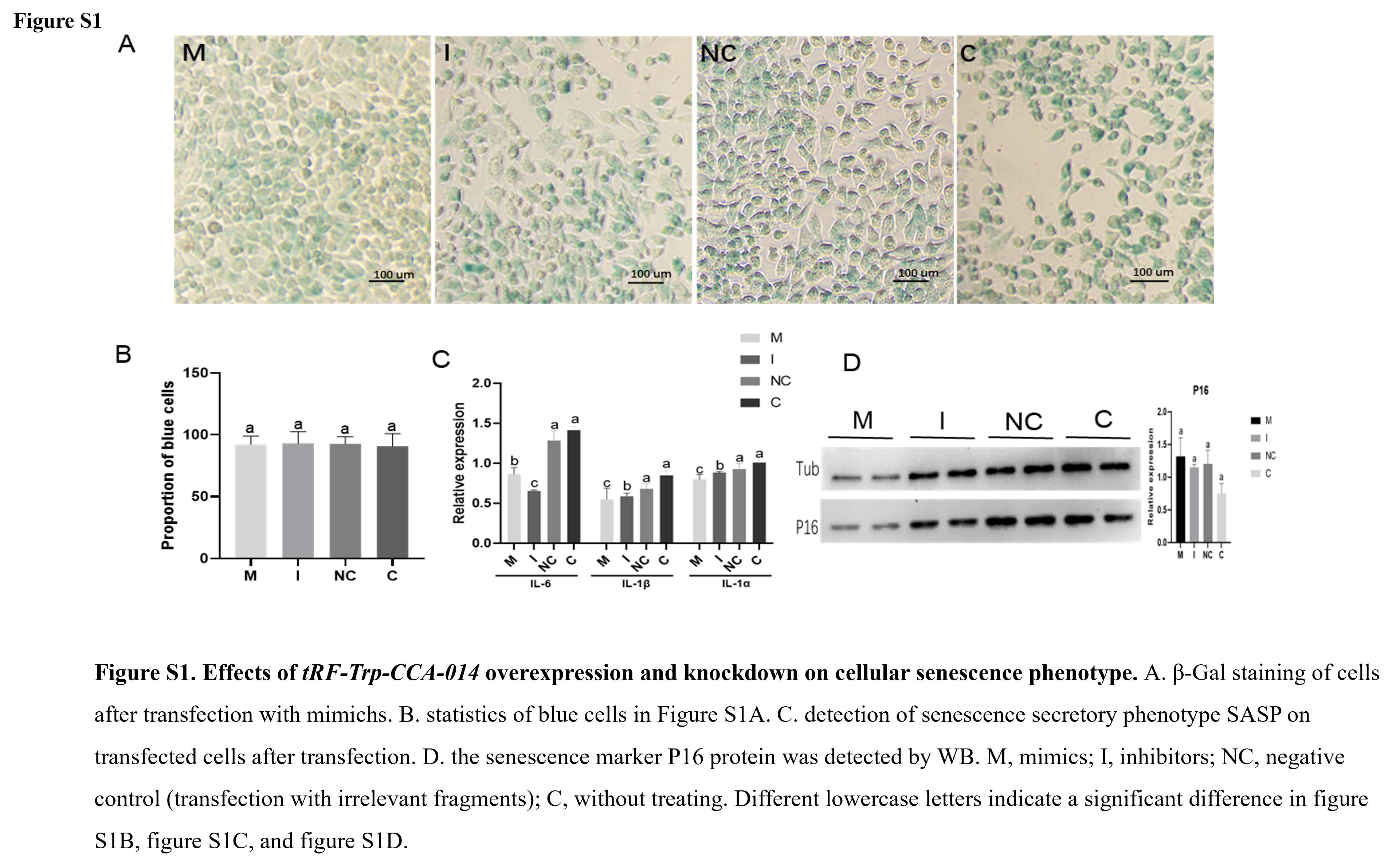

Supplement: Supplementary file 1 [file ijms-24-06169-s001.zip › Figure S1. Effects of tRF-Trp-CCA-014 overexpression and knockdown on cellular senescence phenotype.tif]
